# Assessment of intramyocardial hemorrhage with dark-blood T2*-weighted cardiovascular magnetic resonance

**DOI:** 10.1186/s12968-021-00787-4

**Published:** 2021-07-15

**Authors:** Xingmin Guan, Yinyin Chen, Hsin-Jung Yang, Xinheng Zhang, Daoyuan Ren, Jane Sykes, John Butler, Hui Han, Mengsu Zeng, Frank S. Prato, Rohan Dharmakumar

**Affiliations:** 1 Department of Biomedical Sciences, Cedars-Sinai Medical Center, Biomedical Imaging Research Institute, PACT Bldg – Suite 400, 8700 Beverly Blvd, Los Angeles, CA USA; 2 University of California, Los Angeles, CA USA; 3 Department of Radiology, Zhongshan Hospital, Fudan University, Shanghai, China; 4 Department of Cardiology, Zhongshan Hospital, Fudan University, Shanghai, China; 5 Lawson Health Research Institute, University of Western Ontario, London, ON Canada

**Keywords:** Intramyocardial hemorrhage, T2* MRI, Bright-blood T2*, Dark-blood T2*

## Abstract

**Background:**

Intramyocardial hemorrhage (IMH) within myocardial infarction (MI) is associated with major adverse cardiovascular events. Bright-blood T2*-based cardiovascular magnetic resonance (CMR) has emerged as the reference standard for non-invasive IMH detection. Despite this, the dark-blood T2*-based CMR is becoming interchangeably used with bright-blood T2*-weighted CMR in both clinical and preclinical settings for IMH detection. To date however, the relative merits of dark-blood T2*-weighted with respect to bright-blood T2*-weighted CMR for IMH characterization has not been studied. We investigated the diagnostic capacity of dark-blood T2*-weighted CMR against bright-blood T2*-weighted CMR for IMH characterization in clinical and preclinical settings.

**Materials and methods:**

Hemorrhagic MI patients (n = 20) and canines (n = 11) were imaged in the acute and chronic phases at 1.5 and 3 T with dark- and bright-blood T2*-weighted CMR. Imaging characteristics (Relative signal-to-noise (SNR), Relative contrast-to-noise (CNR), IMH Extent) and diagnostic performance (sensitivity, specificity, accuracy, area-under-the-curve, and inter-observer variability) of dark-blood T2*-weighted CMR for IMH characterization were assessed relative to bright-blood T2*-weighted CMR.

**Results:**

At both clinical and preclinical settings, compared to bright-blood T2*-weighted CMR, dark-blood T2*-weighted images had significantly lower SNR, CNR and reduced IMH extent (all *p* < 0.05). Dark-blood T2*-weighted CMR also demonstrated weaker sensitivity, specificity, accuracy, and inter-observer variability compared to bright-blood T2*-weighted CMR (all *p* < 0.05). These observations were consistent across infarct age and imaging field strengths.

**Conclusion:**

While IMH can be visible on dark-blood T2*-weighted CMR, the overall conspicuity of IMH is significantly reduced compared to that observed in bright-blood T2*-weighted images, across infarct age in clinical and preclinical settings at 1.5 and 3 T. Hence, bright-blood T2*-weighted CMR would be preferable for clinical use since dark-blood T2*-weighted CMR carries the potential to misclassify hemorrhagic MIs as non-hemorrhagic MIs.

## Background

Intramyocardial hemorrhage (IMH) has emerged as an important predictor of adverse long-term outcomes in patients treated with reperfusion therapy for myocardial infarction (MI) [1–5]. Notably, IMH has been associated with delayed infarct healing, larger MIs [6, 7], presence of persistence microvascular obstruction (MVO), higher left ventricular (LV) volumes, compromised LV ejection fraction (LVEF) [6, 8] and late-arrhythmogenic risk [9, 10]. This has precipitated significant clinical interest in the management of MI patients with IMH [11] and driven investigations in the pre-clinical arena focused on understanding the mechanisms contributing to the adverse outcomes.

The reference standard for noninvasive detection and quantification of IMH is T2- and T2*-based cardiovascular magnetic resonance (CMR), which is facilitated by the local distortions in magnetic susceptibility contributing to magnetic field variations mediated by IMH contributing to accelerated decay of the transverse magnetization. While both T2- and T2*-based approaches have been investigated, T2*-based imaging has been shown to have higher sensitivity for detecting and quantifying IMH [12] and the ensuing iron deposition in the chronic MI territories, which appears as hypointense core on T2*-weighted images [7, 13].

Well before the strengths of T2*-based CMR was recognized for IMH detection, T2*-based CMR had become important in the standard of care in patients with global myocardial iron overload diseases such as thalassemia [14]. In this setting, T2*-based CMR was originally performed with bright-blood approaches (with blood in LV chamber appearing bright), but later magnetization-prepared dark-blood T2*-based CMR became common as it offered greater immunity to image artifacts [15–17]. More recently, this notion has also lead to the use of dark-blood prepared T2*-based CMR for the examination of IMH in MI patients [10, 18, 19]. However, the tissue environment of global iron loading and hemorrhagic MI are very different—unlike global iron overloading disorder, in hemorrhagic MI, there is gross increase in edema, localized wall motion abnormalities and only spatially localized increases in iron concentration. Given these differences, it is unclear whether dark-blood T2*-based CMR can yield equivalent diagnostic information as bright-blood T2*-based CMR in the detection and characterization of IMH. We hypothesized that dark-blood T2*-weighted images do not provide equivalent information as bright-blood T2*-weighted images with respect to assessment of IMH. We tested our hypothesis by performing a head-to-head comparison between bright- and dark-blood T2*-weighted CMR in ST-elevation MI patients and validated large animal models with IMH in the acute and chronic phases of MI at 1.5 and 3 T.

## Methods

Both the clinical and animal studies were planned, prospective and randomized as described below.

### Clinical studies—patient selection and imaging protocol

Patient studies were approved by Institutional Review Boards. Following written informed consent, ST-elevation MI (STEMI) patients (n = 29) were enrolled consecutively after successful primary percutaneous coronary intervention (PCI). Patients were randomized for CMR at 1.5 T (n = 14) or 3 T (n = 15). Subsequently the patients underwent CMR scans in the acute phase of MI (7- 10 days post MI). Patients (n = 20) with evidence of IMH on bright-blood T2* CMR were followed up at 6-months post MI with a second CMR. CMRs were performed on 1.5 T (Aera) or 3 T (Verio) CMR systems (Siemens Healthineers, Erlangen, Germany). Following localizers and whole-heart shimming, slice-matched short-axis T2*-weighted acquisitions were performed. All scans were terminated with late-gadolinium enhancement (LGE) CMR. T2*-weighted images were acquired using gradient-recalled acquisitions. Dark-blood T2*-weighted images were acquired with double-inversion-recovery (DIR) preparation applied at the R-wave. All T2*-weighted images were acquired at mid diastole with 7–9 phase encoding lines per heartbeat to minimize motion artifacts. 1.5T: T2*-weighted CMR—number of segments = 9; TR/TE = 1 R-R interval/14.5 ms; flip angle = 18°; bandwidth = 814 Hz/pixel; spatial resolution = 2.0×2.0×8.0 mm^3^; GRAPPA accelerate factor = 2; slice thickness of dark-blood preparation = 200%; inversion time between DIR pulses and readout were between 550 and 700 ms, depending on heart rate. Segmented breath-held LGE images were acquired 10-min post-injection of 0.15 mmol/kg gadolinium contrast agent (Magnevist; Bayer Hearlthcare, Berlin, Germany) using segmented phase-sensitive inversion recovery (PSIR) reconstruction with gradient-recalled-echo readouts (TR/TE = 1 R-R interval/3.2 ms, flip angle = 25°, bandwidth = 140 Hz/pixel, and spatial resolution = 1.3×1.3×8.0 mm^3^). 3T: T2*-weighted CMR—number of segments = 9; TR/TE = 1 R-R interval/12.7 ms; flip angle = 10°; bandwidth = 1030 Hz/pixel; spatial resolution = 1.6 × 1.6×8.0 mm^3^; GRAPPA accelerate factor = 2; and slice thickness of dark-blood preparation = 200%; inversion time between DIR pulses and readout = 550–700 ms, depending on heart rate. LGE images were acquired with TR/TE = 1 R-R interval/1.6 ms; flip angle 20°; bandwidth = 465 Hz/pixel; and spatial resolution = 1.6×1.6×8.0 mm^3^.

### Preclinical studies—animal preparation and imaging protocol

According to the protocol approved by the Institutional Animal Care and Use Committee, hemorrhagic MIs were created in canines (n = 11, all female) by occluding the left-anterior descending coronary artery (LAD) for 3 h, followed by reperfusion [7]. Prior to CMR scans, all animals were intubated and anesthetized with isoflurane (1–1.5%/volume). All animals were studied in a 3 T CMR system (Verio, Siemens Healthineers) in the acute phase (7 days post reperfusion) and in the chronic phase (> 2 months post reperfusion). A subset of animals (n = 8) were also studied at 1.5 T (Aera, Siemens Healthineers) in the acute phase and the chronic phases post MI. Slice-matched, breath-held, electrocardiogram (ECG) triggered, bright-blood T2*-weighted and DIR-prepared dark-blood T2*-weighted images were acquired. All T2*-weighted images were acquired at mid diastole with 7–9 phase encoding lines per heartbeat to minimize motion artifacts. At 1.5 T, T2*-weighted images were acquired with TR/TE = 1 R-R interval/14.4 ms; segments = 7; flip angle = 20°; bandwidth = 815 Hz/pixel; and spatial resolution = 1.1×1.1×6.0mm^3^; GRAPPA accelerate factor = 2; slice thickness of dark-blood preparation = 200%, inversion time between DIR pulses and readout = 500 to 700 ms. Scan parameters for LGE were TR/TE = 1 R-R interval/3.3 ms, flip angle = 20°, bandwidth = 235 Hz/pixel, spatial resolution = 1.1×1.1×6.0 mm^3^. At 3 T, T2*-weighted images were acquired with TR/TE = 1 R-R interval/11.5 ms; segments = 7; flip angle = 18°; bandwidth = 925 Hz/pixel; and spatial resolution = 1.1 × 1.1×6.0mm^3^; GRAPPA accelerate factor = 2; slice thickness of dark-blood preparation = 200%, inversion time between DIR pulses and readout = 500 to 700 ms. LGE images were acquired at 3 T with TR/TE = 1 R-R interval/2.1 ms, flip angle = 20°, bandwidth = 287 Hz/pixel, spatial resolution = 1.1×1.1×6.0 mm^3^.

Following CMR scans in the chronic phase, animals were euthanized, hearts were explanted. Nine of the hearts were fixed in 10% formalin solution and scanned at 3 T for ex-vivo imaging. 3D T2*-weighted images were acquired with TR/TE = 16.0/11.5 ms and spatial resolution = 1.0× 1.0×1.5 mm^3^. Two hearts were cut into 10 mm-thick short axis rings and representative samples of infarcted and remote myocardium were cut and embedded in paraffin. These sections were stained with Prussian blue stain to confirm presence or absence of iron.

### Image analyses—image quality, hemorrhage extent and diagnostic performance

#### Hemorrhage detection

All image analyses were performed with cvi42 (Circle Cardiovascular Imaging, Calgary, Alberta, Canada) by two expert readers and the results were averaged unless stated otherwise. Remote myocardium was identified as the region absent of hyperintensity on LGE images. MI zone was defined as the region with mean signal intensity (SI) of at least 5 standard deviations (SD) greater than that of a reference region-of-interest (ROI) drawn in remote myocardium [20]. MI zones were identified to be hemorrhagic if there were hypointense cores within MI on the bright-blood T2*-weighted images (TE = 14.5 ms (patients) 14.4 ms (animals) at 1.5 T and 12.7 ms (patients) and 11.5 ms (animals) at 3 T) with a mean signal intensity 2-SD lower than that of the reference ROI in the remote myocardium [7, 13]. A TE of ~ 14 ms at 1.5 T and ~ 12 ms at 3 T were chosen to balance the image contrast and image artifacts based on previous reports [7, 21]. IMH volume determined from each heart based on T2*-weighted images were normalized by the total LV myocardial volume and reported as IMH Extent.

#### Signal characteristics

SI values of IMH regions and remote myocardium determined from T2*-weighted images were used to compute signal-to-noise ratio (SNR), contrast-to-noise ratio (CNR). Relative SNR and Relative CNR were computed and reported as:

1 $$Relative~SNR~ = ~\frac{{SI\_remote_{{DB}} /\sigma \_air_{{DB}} }}{{SI\_remote_{{BB}} /\sigma \_air_{{BB}} }} \times 100\%$$

2 $$Relative~CNR~ = ~\frac{{(SI\_remote_{{DB}} - SI\_IMH_{{DB}} )/\sigma \_air_{{DB}} }}{{(SI\_remote_{{BB}} - SI\_IMH_{{BB}} )/\sigma \_air_{{BB}} }} \times 100\%$$

where $SI\_remote$ is the mean intensity of remote myocardium, $SI\_IMH$ is mean intensity of IMH region and $\sigma \_air$ is the SD of signal intensity of background air. DB is dark-blood and BB is bright-blood.

Coefficient of variations (COV) were computed as:

3 $$COV~ = ~~\frac{\sigma }{{SI}}$$

where $SI$ is the mean signal intensity of the region of interest, and $\sigma$ is the SD of SI of the ROI.

#### Diagnostic performance—sensitivity, specificity, accuracy and interobserver variability

Sensitivity and specificity of dark-blood T2*-weighted images for detection of IMH for each subject were determined with bright-blood T2*-weighted images serving as the ground truth based on previous work [13] and findings here. All bright-blood T2*-weighted images were segmented according to the recommendation of American Heart Association (AHA). Segments were considered positive for IMH if the hypointense area exceeded 1% of the cross-sectional area of the segment. Segments affected by off-resonance, particularly near the heart–lung interface, were manually excluded. Accuracy was computed as a quotient of number of true positives and true negatives normalized by the total number of segments evaluated. The interobserver variability in IMH Extent was determined based on the independent assessment by the two expert readers.

### Statistical analyses

Statistical analysis was performed using SPSS (version 23, Statistical Package for the Social Sciences, International Business Machines, Inc., Armonk, New York). Normality of continuous data was determined by using the Shapiro–Wilk test and quantile–quantile plots. Normally distributed variables were compared using repeated measures ANOVA. Repeated measures from each heart were nested for analysis. Pairwise comparisons for normal data were performed using paired t-test, and for non-normal data were performed using the Mann–Whitney *U* test. Inter-observer reliability in measuring IMH Extent was determined using intraclass correlation coefficient. Bland–Altman analysis of IMH Extent determined using dark- and bright-blood T2*-weighted images was performed to determine the bias in measurements. IMH Extent determined using in-vivo T2*-weighted images and using ex-vivo T2*weighted images were regressed against one another to assess the correlation between in-vivo and ex-vivo T2*-weighted images. Statistical significance was set at *p* < 0.05.

## Results

From the 29 patients undergoing CMR following acute STEMI, a total of 20 patients (17 male, 34–65 years, 58 to 92 kg) were identified to be positive for IMH and 10 patients were assigned to the 1.5 T group and the remaining 10 were assigned to the 3 T group. At the 6-month follow up, the same patients were studied at the respective field strengths. From the 1.5 T studies, 31 slices were positive for IMH in the acute phase and 21 were positive for iron in the chronic phase. From the 3 T studies, 28 slices were positive for IMH in the acute phase and 17 were positive for iron in the chronic phase. A few slices were discarded from further analysis due to off-resonance artifacts (1.5 T: 3 in acute phase and 2 in chronic phase; and at 3 T: 1 slice from acute phase; none from chronic phase).

All animals survived hemorrhagic MI and were studied at 3 T (n = 11), and a subset of the same animals were also studied at 1.5 T (n = 8). From the 1.5 T studies, 27 slices were positive for IMH in the acute phase and 21 slices were positive for iron in the chronic phase. From the 3 T data sets, 39 slices were positive for IMH in the acute phase and 29 were positive for iron in the chronic phase. From these data sets, 1 slice from acute phase was removed from further analysis due to off-resonance artifacts at 3 T.

### Case examples

**Fig. 1:**
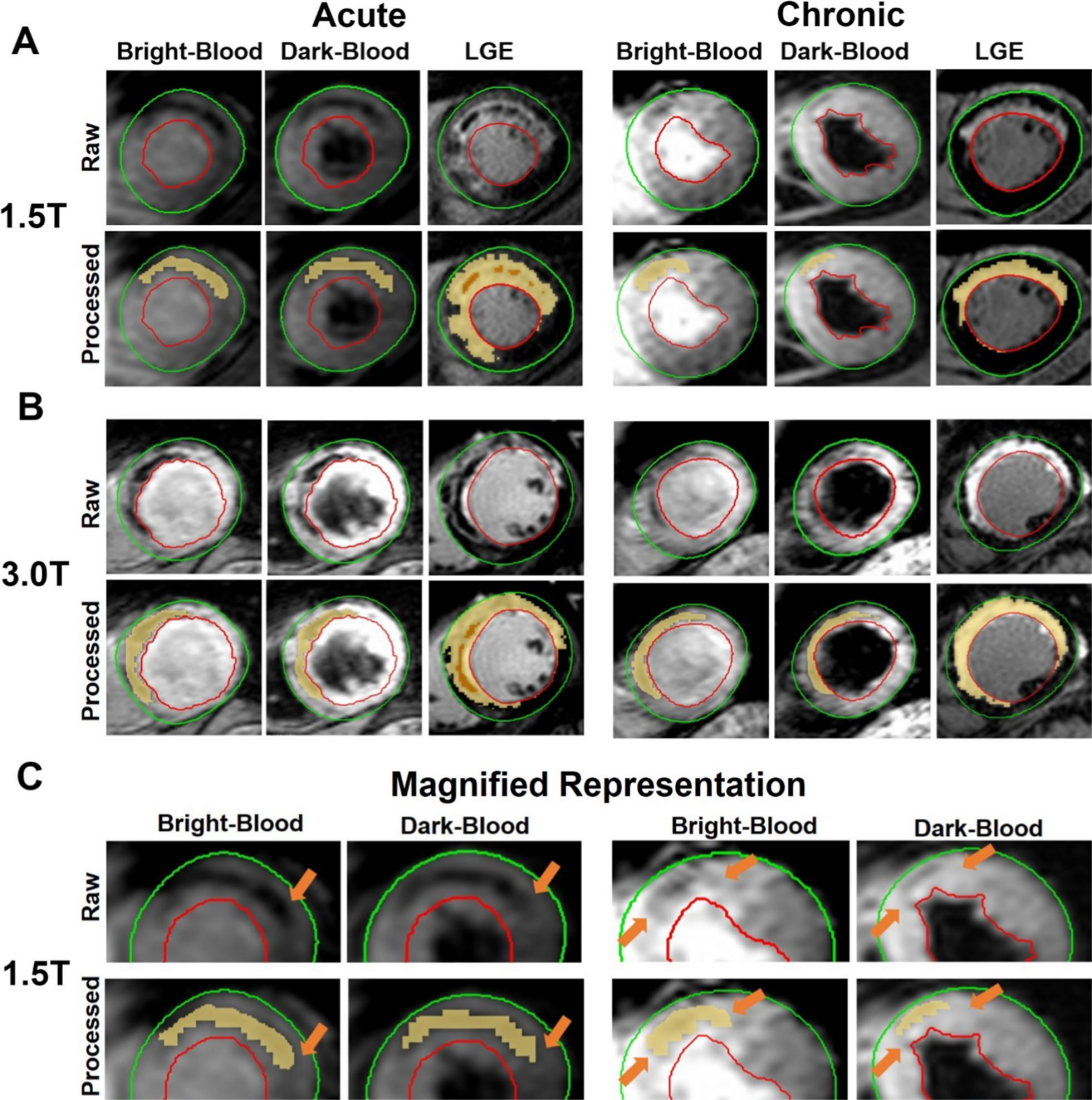
Bright-blood vs. dark-blood T2*-weighted cardiovascular magnetic resonance (CMR) in hemorrhagic myocardial infarction (MI) patients. Representative bright- and dark-blood T2*-weighted and late gadolinium enhancement (LGE) images acquired from patients with hemorrhagic MIs in the acute and chronic phases of MI at 1.5 T (55 year-old male; **A**) and 3 T (42 year-old male; **B**) are shown. Panel **C** is magnified representation of the Intramyocardial hemorrhage (IMH) detected on bright-blood and dark-blood T2*-weighted images at 1.5 T in acute and chronic phases. Arrows point to the regions where hypointensity is seen in bright-blood but not in dark-blood images

Representative bright- and dark-blood T2*-weighted and LGE images acquired at 1.5 and 3 T in the acute and chronic phases in patients with hemorrhagic MI are shown in Fig. 1. Note that although the IMH region is accurately identified (as the region with mean signal intensity at least 2-SD lower than that of remote myocardium) in the dark-blood T2*-weighted image, in relation to the bright-blood T2*-weighted image, the extent of hemorrhage is visually smaller independent of MI age or field strength.

**Fig. 2:**
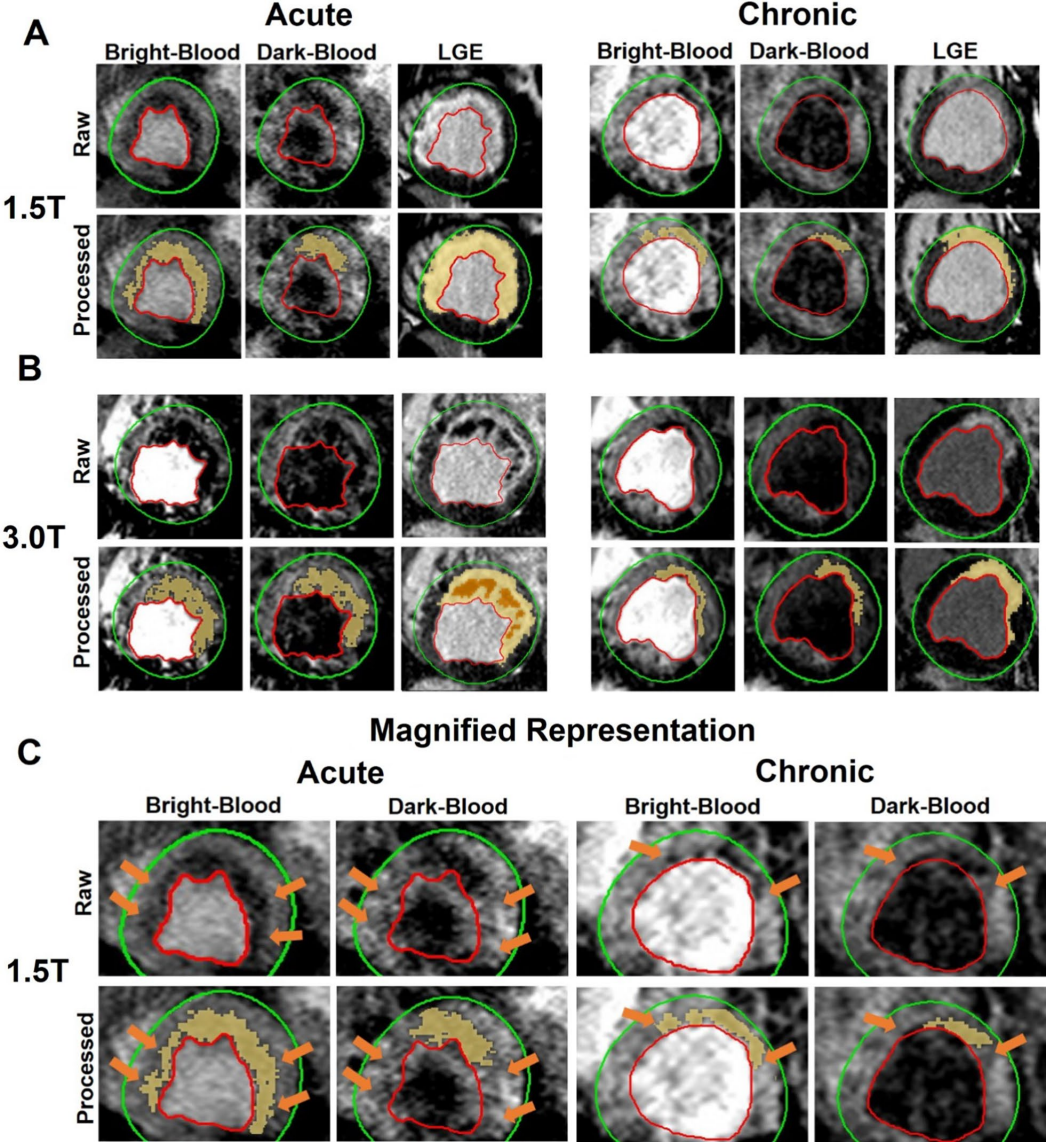
Bright-blood vs. dark-blood T2*-weighted CMR in canines with hemorrhagic MI. Representative bright- and dark-blood T2*-weighted and LGE images from canines with hemorrhagic MI in the acute and chronic phase of MI at 1.5 T (**A**) and 3 T (**B**) are shown. Both raw and processed (details in text) images are shown with the processed images delineating the regions of hemorrhage (T2*-weighted images) and MI (LGE) territories. **C** is magnified representation of the IMH detected on bright-blood and dark-blood T2*-weighted images at 1.5 T in acute and chronic phases. Arrows point to the regions where hypointensity is seen in bright-blood but not in dark-blood images

Representative bright- and dark-blood T2*-weighted and LGE images acquired at 1.5 and 3 T in the acute and chronic phases in canines with hemorrhagic MI are shown in Fig. 2. Similar to the patient data in Fig. 1, the extent of IMH is significantly smaller in dark-blood images independent of MI age and imaging field strength.

### Relative SNR, relative CNR and COV: Dark-blood vs. bright-blood T2*-weighted CMR

**Fig. 3:**
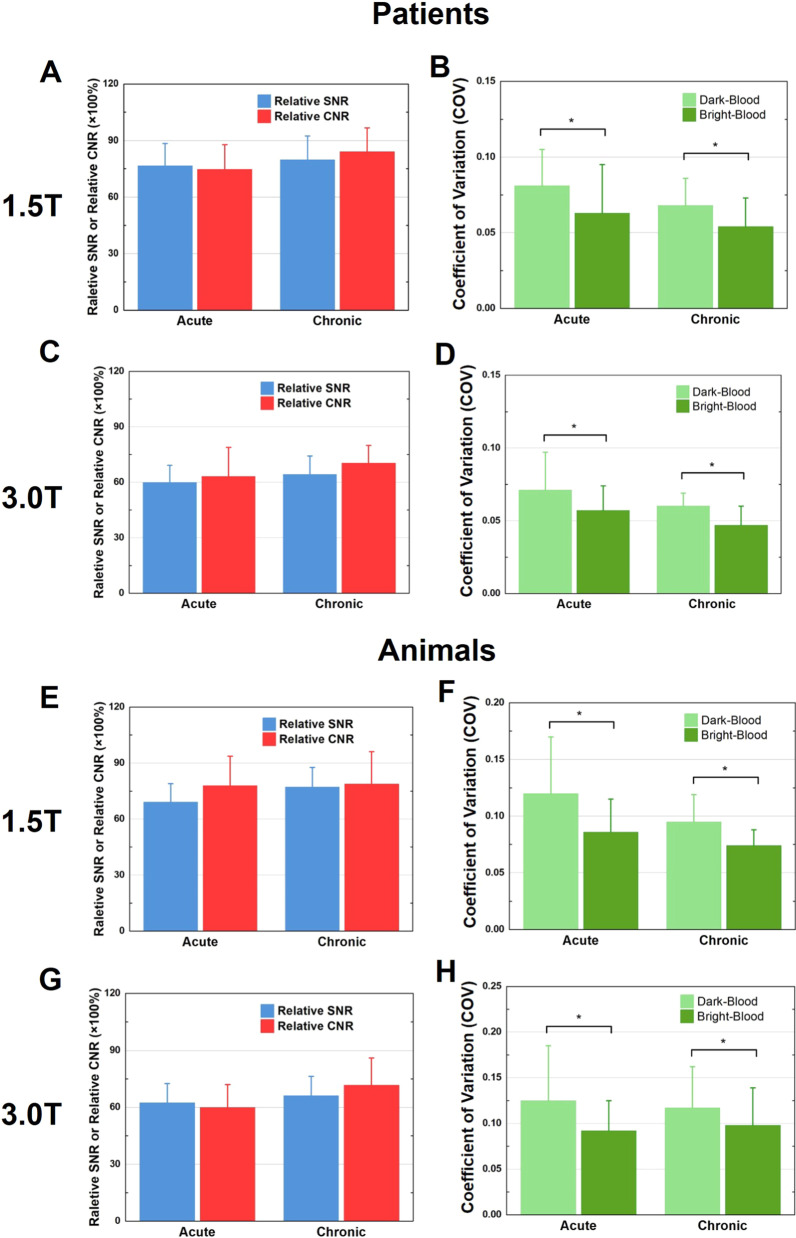
Effect of dark-blood magnetization preparation on T2*-weighted signal characteristics. Relative signal-to-noise (SNR), Relative contrast-to-noise (CNR) and coefficient of variations (COV) computed from T2*-weighted images in patients at 1.5 T (**A**, **B**, respectively) and 3 T (**C**, **D**, respectively) and animals at 1.5 T (**E**, **F**, respectively) and 3 T (**G**, **H**, respectively) in the acute and chronic phases of hemorrhagic MI are shown. All Relative SNR and Relative CNR were found to be less than 100 (*p* < 0.05); and *denotes that the measures being compared are statistically different (*p* < 0.05)

The appearance of hemorrhage was evidenced as hypointense core on both bright- and dark-blood T2*-weighted images at 1.5 and 3 T, in the acute and chronic phases of MI. Relative SNR, relative CNR and COV relations between dark- and bright-blood T2*-weighted images are shown in Fig. 3.

Mean relative SNR of remote myocardium from bright- and dark-blood T2*-weighted images at 1.5 and 3 T in the acute and chronic phases of MI are shown in (Fig. 3A and C). The relative SNR of remote myocardium between the dark-blood and bright-blood T2*-weighted images were 76.6 ± 11.8% (*p* < 0.05) in acute phase and 79.7 ± 12.7% (*p* < 0.05) in chronic phase at 1.5 T. At 3 T, the relative SNR between the dark-blood and bright-blood T2*-weighted images was even lower (acute phase: 60.0 ± 9.1%, p < 0.05; chronic phase: 64.2 ± 10.0%, *p* < 0.05). Similar observations were evident in animals as well (Fig. 3, E and G). Relative SNR values were 69.1 ± 9.9% (*p* < 0.05) in acute phase and 77.2 ± 10.5% (*p* < 0.05) in chronic phase at 1.5 T. And at 3 T, relative SNR in acute phase is 62.3 ± 10.2% (*p* < 0.05), in chronic phase is 66.2 ± 10.1% (*p* < 0.05).

Mean relative CNR between IMH and remote myocardium at 1.5 and 3 T in patients are shown in (Fig. 3A and C). Compared to bright-blood T2*-weighted images, dark-blood T2*-weighted images showed significantly lower CNR at 1.5 T. Relative CNR values are 74.7 ± 13.1% (*p* < 0.05) in acute phase and 84.1 ± 12.6% (*p* < 0.05) in chronic phases of MI. Similar observations were found at 3 T as well, with relative CNR values are 63.1 ± 15.8% (*p* < 0.05) in the acute and 70.3 ± 9.6% (*p* < 0.05) in the chronic phases of MI. Results from animals (Fig. 3E and G) were consistent with patient studies. In animals, the relative CNR at 1.5 T was 77.9 ± 15.8% (*p* < 0.05) in acute phase; and 78.8 ± 17.3% (p < 0.05), in chronic phase; and at 3 T, 59.8 ± 12.2% (*p* < 0.05) in acute phase; and 71.4 ± 14.4% (*p* < 0.05) in chronic phase.

COV of remote myocardium at 1.5 T and 3 T in patients are shown in (Fig. 3B and D). COV was higher in dark-blood T2*-weighted images than that on bright-blood T2*-weighted images in general. At 1.5 T, COV was 32.1 ± 65.0% (*p* < 0.05) and 27.1 ± 55.1% (*p* < 0.05) higher on dark-blood T2*-weighted images in acute and chronic phase respectively. At 3 T, COV was 26.5 ± 30.2% (*p* < 0.05) greater in the acute phase and 38.7 ± 52.3% (*p* < 0.05) greater in the chronic phase of MI. Similar results were found in animals (Fig. 3F and H). COV was 49.4 ± 65.2% (*p* < 0.05) greater on dark-blood T2*-weighed images than that on bright-blood T2*-weighted images in the acute phase of MI and 33.0 ± 41.8% (*p* < 0.05) greater in the chronic phase of MI at 1.5 T. At 3 T, COV increased by 35.6 ± 40.0% (*p* < 0.05) in the acute phase and by 37.0 ± 55.3% (*p* < 0.05) in the chronic phase of MI.

### Quantification of IMH extent: dark-blood vs. bright-blood T2*-weighted CMR

**Fig. 4:**
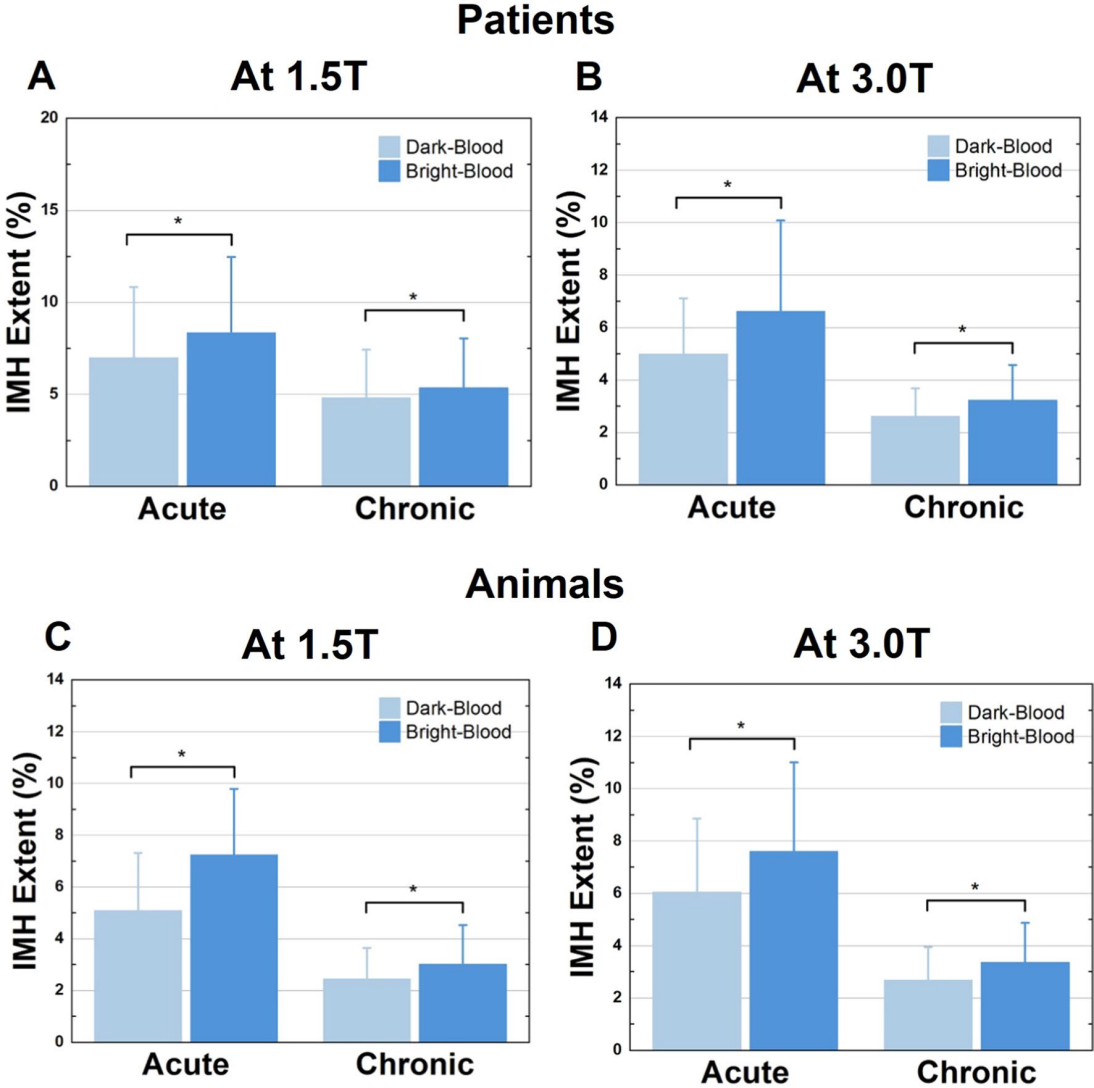
Impact of dark-blood preparation on IMH extent determined from T2*-weighted CMR in patients and animals. IMH extent in patients is underestimated by dark-blood-prepared T2*-weighted images at 1.5 T and 3 T (**A**, **B**, respectively). IMH extent in animals is underestimated by dark-blood-prepared T2*-weighted images at 1.5 T and 3 T (**C**, **D**, respectively). *denotes statistically significant difference (*p* < 0.05) between bright- and dark-blood images

**Fig. 5:**
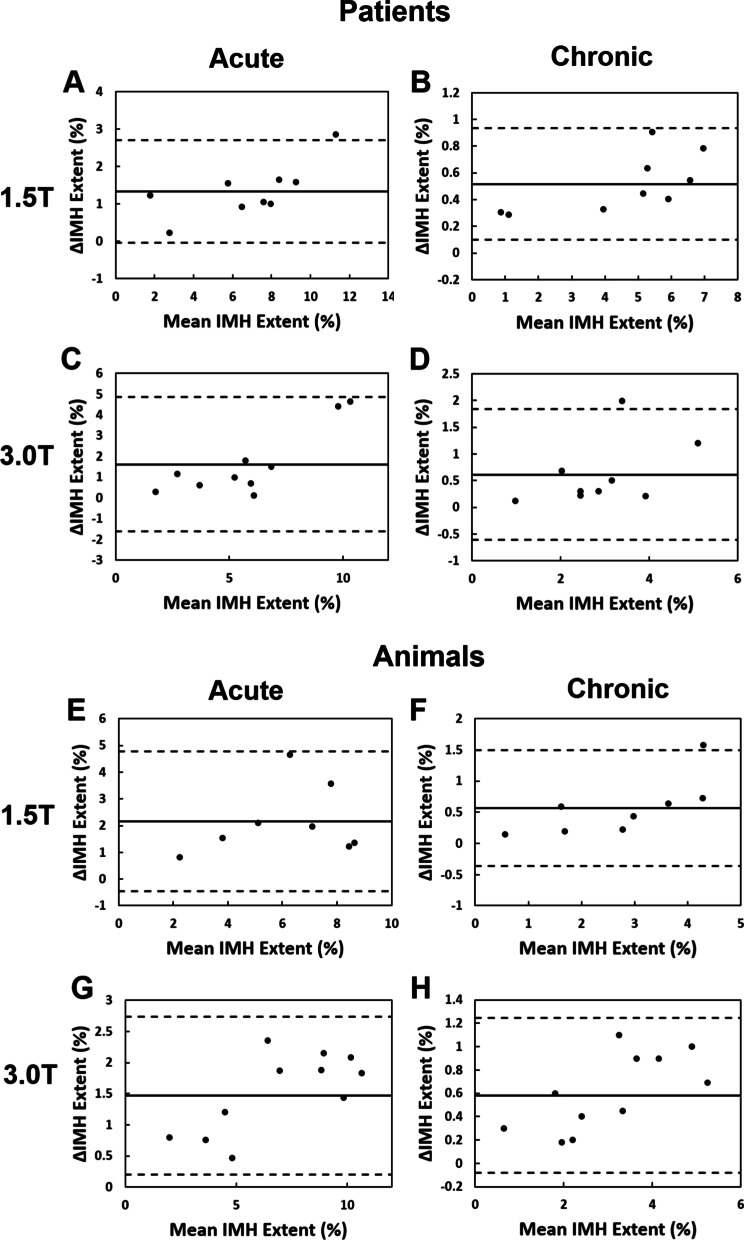
Bland–Altman plots of IMH extent determined from bright- and dark- blood T2*-weighted CMR in patients and animals. Moderate bias in IMH extent was found between bright- and dark- blood T2*-weighted images in patients and animals at 1.5 T and 3 T in the acute and chronic phases of MI

Consistent with the reduction in SNR and CNR and amplification on COV of the remote myocardium, IMH extent was significantly smaller on dark-blood T2*-weighted images compared to bright-blood T2*-weighted images (Fig. 4), independent of field strength or age of MI in both patients and animals. In patients at 1.5 T, IMH extent in dark-blood T2*-weighted was reduced by 18.7 ± 12.9% (p < 0.05) in acute phase and by 12.7 ± 8.1% (*p* < 0.05) in the chronic phase of MI relative to bright-blood T2*-weighted images. At 3 T, the IMH extent on dark-blood T2*-weighted images were reduced by 21.6 ± 11.8% (*p* < 0.05) in the acute phase and by 17.4 ± 12.6% (*p* < 0.05) in the chronic phase compared to bright-blood T2*-weighted images. In animals, at 1.5 T, IMH extent measured from dark-blood T2*-weighted images were reduced by 30.2 ± 13.1% (*p* < 0.05) and by 23.2 ± 11.0% (*p* < 0.05) compared the corresponding bright-blood T2*-weighted images in the acute and chronic phases, respectively. At 3 T, the IMH extent on dark-blood T2*-weighted images were reduced by 21.3 ± 6.9% (*p* < 0.05) in the acute phase and 20.6 ± 12.1% (*p* < 0.05) in the chronic phase compared to the corresponding bright-blood T2*-weighted images. Bland–Altman plots with 95% limits of agreement (Fig. 5) showed a modest bias between the IMH extent between the two approaches as well. A bias of 1.3 ± 1.3% was found between dark- and bright-blood T2*-weighted images of IMH extent at 1.5 T in acute phase of MI and 0.5 ± 0.4% in chronic phase of MI in patients. At 3 T, the bias between the methods was 1.6 ± 3.2% in the acute phase and 0.6 ± 1.2% in the chronic phase of MI. In animal studies, the bias in IMH extent determined from dark- and bright -blood T2*-weighted images was 2.2 ± 2.6% in the acute phase and 0.6 ± 0.9% in the chronic phase at 1.5 T; and 1.5 ± 1.2% in acute phase and 0.6 ± 0.7% in chronic phase of MI at 3 T.

### Qualitative observations of compromised image quality on dark-blood T2*-weighted detection of IMH

**Fig. 6:**
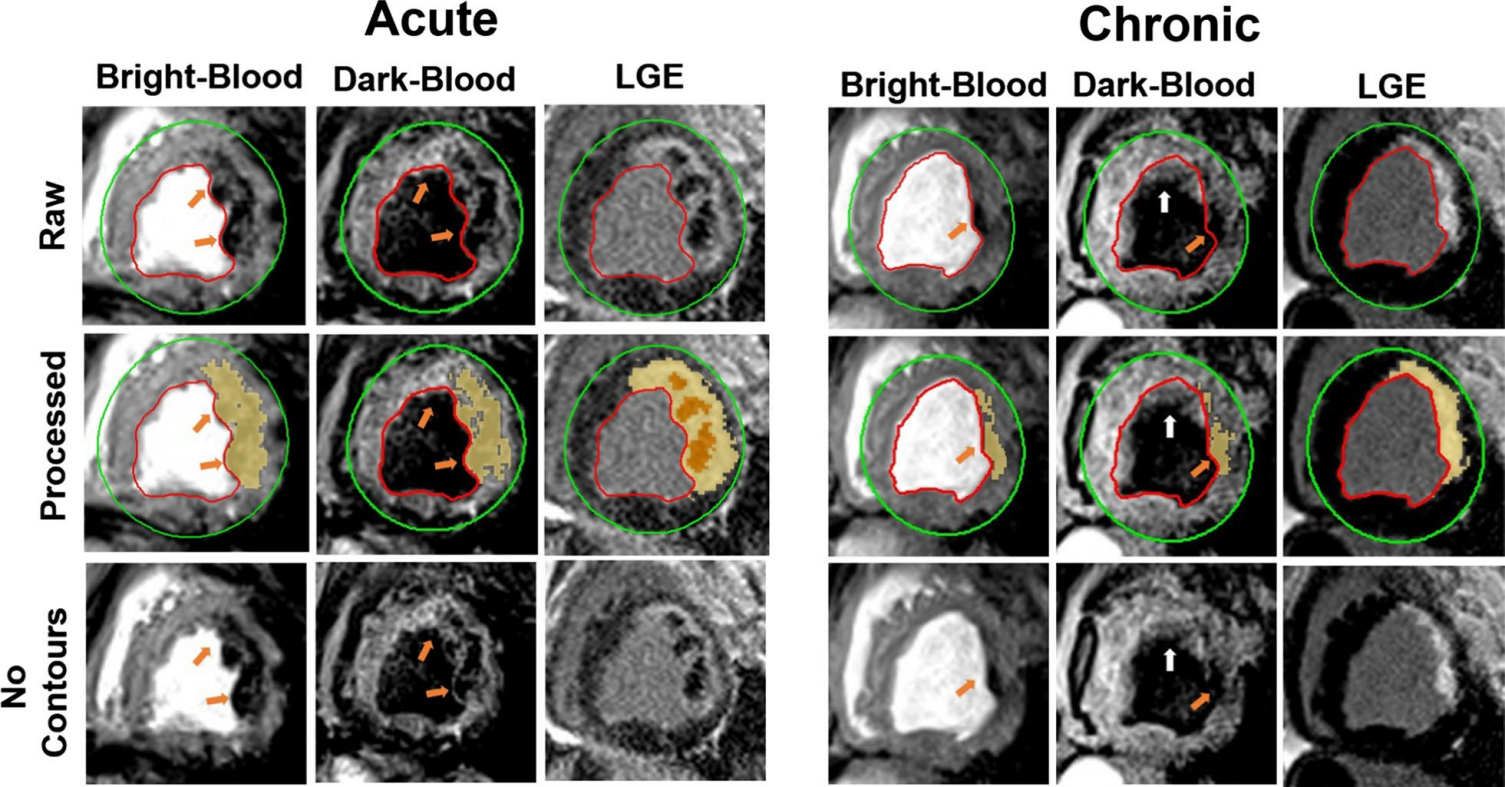
Qualitative differences in dark-blood vs. bright-blood T2*-weighted CMR. A representative case from a canine with acute IMH and chronic ensuing iron deposition demonstrating evidence of stagnant blood in the MI zone and hypointense appearance of IMH, both contributing to the compromised visual delineation of IMH on dark-blood T2*-weighted CMR compared to bright-blood T2*-weighted CMR

Example bright- and dark-blood T2*-weighted and LGE images obtained from an infarcted animal with IMH in the acute and chronic phase of MI are shown in Fig. 6. A common observation in dark-blood T2*-weighted images (as visualized in Fig. 6) was the appearance of stagnant blood obscuring the boundary between blood and myocardium, likely from compromised contraction of the infarct wall. Another key difficulty observed with dark-blood T2*-based imaging is that IMH appearing hypointense makes it difficult to visually appreciate the presence of IMH (or residual iron) when it is found in the subendocardial wall (Table 1).

**Table 1:** Clinical features of patients (n = 20)

Parameter	1.5 T (n = 10)	3 T (n = 10)
Age (years)	55 (34–65)	54 (42–65)
Male Sex	9	8
Weight (kg)	68.5 (58–82)	70.9 (61–85)
Infarct-related coronary artery	LAD (n = 9)	LAD (n = 7)
	LCX (n = 0)	LCX (n = 2)
	RCA (n = 1)	RCA (n = 1)
Time to reperfusion (hrs), median (IQR)	7.5 (4–18)	6.0 (4–8)
Modality of reperfusion	PCI (n = 10)	PCI (n = 10)
Heart rate (beats per minute)	84 (64–90)	85 (75–108)
Antiplatelet (dual) therapy	10	10
MVO volume (%LV)	9.1 ± 6.3	7.5 ± 5.8

*LAD* Left-anterior descending coronary artery, *LCX* Left-circumflex coronary artery, *MVO* Microvascular obstruction, *PCI* Percutaneous coronary intervension, *RCA* Right coronary artery

### Inter-observer variability: dark-blood vs. bright-blood T2*-weighted CMR

Inter-observer variability in IMH Extent measured by two expert readers is reported as intraclass correlation coefficients with 95% confidence interval in Table 2. In both patients and animals, there was good to excellent agreements in IMH extent determined by the two expert reviewers when bright-blood T2*-weighted images were used. However, dark-blood T2*-weighted images, although lead to modest to good agreement, they performed consistently weaker compared to bright-blood T2*-weighted images with respect to IMH extent.

**Table 2:** Inter-observer variability in quantifying IMH extent with dark-blood and bright-blood T2*-weighted CMR

		MI Age	Dark blood	Bright blood
Patients	1.5 T	Acute	0.790 (0.345–0.932)	0.903 (0.697–0.969)
		Chronic	0.756 (− 0.544–0.965)	0.809 (− 0.898–0.980)
	3 T	Acute	0.842 (0.358–0.957)	0.922 (0.726–0.979)
		Chronic	0.640 (− 0.275–0.916)	0.801 (− 0.230–0.968)
Animals	1.5 T	Acute	0.813 (− 0.180–0.956)	0.933 (0.555–0.982)
		Chronic	0.688 (0.104–0.894)	0.843 (− 0.162–0.965)
	3 T	Acute	0.849 (0.026–0.969)	0.935 (0.533–0.987)
		Chronic	0.607 (− 0.233–0.895)	0.812 (− 0.169–0.965)

Numbers reported in parenthesis represent the 95% confidence interval. *MI* Myocardial infarction

### Validation of bright-blood T2*-weighted imaging comparing to ex-vivo T2*-weighted imaging

**Fig. 7:**
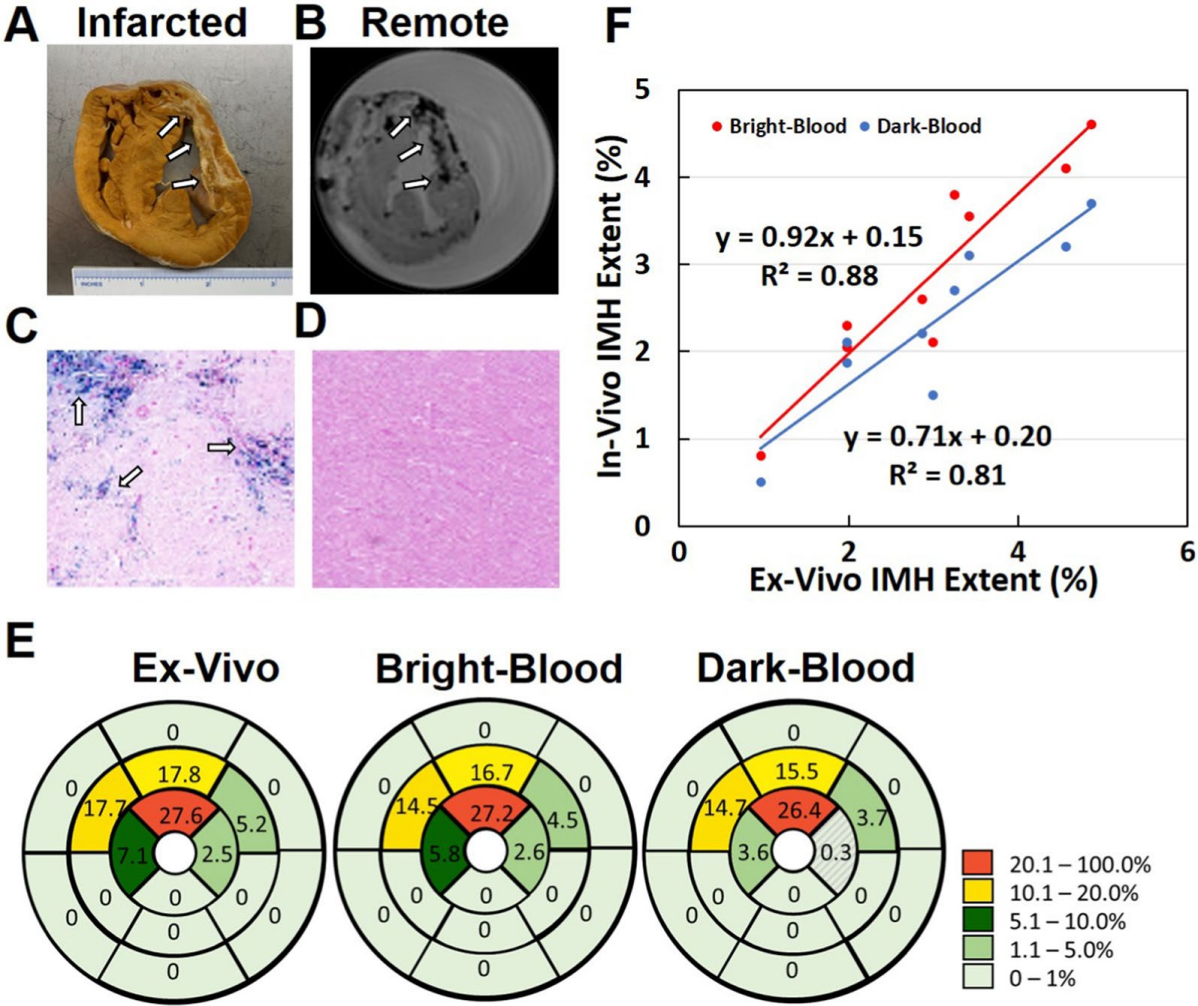
Ex-vivo validation of T2*-weighted CMR for detection of intramyocardial hemorrhage. A short-axis view of a formalin fixed heart from an animal captured with a photograph (**A**) and 3 T ex-vivo CMR (**B**) in the chronic phase MI from one animal are shown. Arrows point to chronic MI territories with history of hemorrhagic MI. Paraffin-fixed myocardial sections (infarcted and remote) stained with Prussian blue from an animal are shown (**C**, **D**). Note that the infarcted regions show evidence of iron (blue stains, arrow heads, **C**) consistent with history of hemorrhagic infarction, which is not evident in the remote territory (**D**). **E** shows the American Heart Association segmentation with IMH extent within each segment. Generally, there was good agreements of segmental IMH extent between ex-vivo segments and bright- and dark-blood images. However, one of the segments in dark-blood T2*-weighted images was not identified to be positive for IMH (< 1%) relative to the ex-vivo and bright-blood T2*-weighted images. **F** shows strong correlation of IMH extent (as fraction of whole LV volume) between in-vivo bright-blood T2*-weighted CMR and ex-vivo T2*-weighted CMR across all animals (y = 0.92x + 0.15, R^2^ = 0.88, *p* < 0.05). The same panel also shows very good correlation between in-vivo dark-blood T2*-weighted CMR and ex-vivo T2*-weighted CMR across all animals (y = 0.71x + 0.20, R^2^ = 0.81, *p* < 0.05)

A short-axis view of a formalin fixed heart from an animal and 3 T ex-vivo CMR in the chronic phase MI from one animal are shown in (Fig. 7A, B). Paraffin-fixed sections of the heart stained with Prussian blue are shown in (Fig. 7C, D). Hemorrhage was confirmed by the evidence of iron. Corresponding AHA segmentation of IMH in chronic phase of MI from ex-vivo T2*-weighted, bright-blood T2*-weighted and dark-blood T2*-weighted images at 3 T is shown in (Fig. 7E). Relative to ex-vivo T2*-weighted CMR, sensitivity and specificity for detection of IMH based on bright-blood T2*-weighted images were both 100%, which was not the case with dark-blood T2*-weighted CMR. Across all animals, IMH extent computed from in-vivo bright-blood T2*-weighted CMR and ex-vivo T2*-weighted CMR were strongly correlated (See Fig. 7 F). Similarly, very good correlation was found between dark-blood T2*-weighted CMR and ex-vivo T2*-weighted CMR (Fig. 7F), albeit the agreement was slightly weaker compared to bright-blood T2*-weighted CMR.

### Diagnostic performance: dark-blood vs. bright-blood T2*-weighted CMR

Table 3 shows sensitivity, specificity, accuracy and AUC for detection of IMH in patients and animals based on dark-blood T2*-weighted images, with bright-blood T2*-weighted images serving as the ground truth. In patients, at 1.5 T, dark-blood T2*-weighted images showed moderate sensitivity, good specificity and moderate accuracy for detection of IMH in acute phase of MI; and moderate sensitivity, specificity and accuracy in chronic phase for detection of residual iron in the chronic phase of hemorrhagic MI. At 3 T, dark-blood T2*-weighted images showed moderate sensitivity, good specificity and accuracy for detection of hemorrhage in acute phase of MI, and good sensitivity, specificity and accuracy in chronic phase for detection of residual iron in the chronic phase of hemorrhagic MI. In animals, at 1.5 T, dark-blood T2*-weighted images showed excellent sensitivity, moderate specificity and good accuracy for detection of IMH in acute phase of MI; and moderate sensitivity, specificity and accuracy in chronic phase for detection of residual iron in the chronic phase of hemorrhagic MI. At 3 T, dark-blood T2*-weighted images showed excellent sensitivity, good specificity and accuracy for detection of hemorrhage in acute phase of MI, and moderate sensitivity, specificity and accuracy in chronic phase for detection of residual iron in the chronic phase of hemorrhagic MI.

**Table 3:** Diagnostic performance of dark-blood T2*-weighted MRI for detecting IMH

		Age of MI	Sensitivity (%)	Specificity (%)	Accuracy (%)	AUC
Patients	1.5 T	Acute	82.6 ± 15.8*	95.7 ± 8.1	87.5 ± 9.4*	0.892 ± 0.091*
		Chronic	83.7 ± 13.9*	93.4 ± 10.7	83.3 ± 11.3*	0.886 ± 0.098*
	3 T	Acute	73.5 ± 20.0*	94.7 ± 8.2	88.8 ± 8.0*	0.841 ± 0.138*
		Chronic	87.1 ± 21.6*	92.6 ± 10.1	91.3 ± 10.2*	0.899 ± 0.137*
Animals	1.5 T	Acute	88.1 ± 8.0*	94.1 ± 6.8	92.9 ± 5.8*	0.911 ± 0.068*
		Chronic	86.1 ± 10.6*	94.4 ± 11.0	90.4 ± 11.8*	0.903 ± 0.128*
	3 T	Acute	89.6 ± 9.0*	95.0 ± 8.7	91.6 ± 7.2*	0.923 ± 0.006*
		Chronic	85.7 ± 12.9*	92.9 ± 18.8	89.7 ± 12.9*	0.893 ± 0.142*

^*^denotes *p* < 0.05

## Discussion

Intramyocardial hemorrhage, which can be noninvasively detected using T2*-based CMR, has emerged as one of the strongest predictors of adverse outcome in post MI patients [6–8, 10, 22]. However, likely driven by the general consensus [17] in the field that dark-blood T2* CMR is preferable over bright-blood T2* CMR for imaging cardiac iron overload (such as in thalassemia), a number of recent studies have adopted dark-blood T2* CMR for imaging IMH [10, 18, 19]. To date however, only bright-blood T2*-based CMR has been validated for imaging IMH [13, 21] and the relative performance of dark-blood T2*-based CMR against bright-blood T2*-based CMR for IMH detection is not known. To address this gap, we investigated the relative performance of dark-blood T2*-weighted CMR against bright-blood T2*-weighted CMR for imaging IMH. To this end, we examined the image characteristics and diagnostic performance of dark-blood-prepared T2*-weighted CMR against bright-blood T2*-weighted CMR in MI patients and large animal models with IMH in the acute and chronic phases at 1.5 and 3 T. Independent of subject cohort studied (patients or animals with hemorrhagic MI), field strength (1.5 or 3 T) and age of MI (acute or chronic MI), we found that SNR and CNR were significantly lower in dark-blood T2*-weighted CMR compared to the bright-blood counterpart. We also found that the variability of signal in T2*-weighted images to be greater in dark-blood prepared images compared to the bright-blood images. Notably, we found that IMH Extent, characterized as relative size of IMH to LV, was reduced in dark-blood T2*-weighted images compared to bright-blood T2*-weighted images. The observations on the reduced IMH extent is consistent with the observed loss in SNR and CNR in the dark-blood images. As discussed later, these discrepancies likely also facilitated a compromise in the diagnostic performance of dark-blood T2*-based CMR for classifying MIs as hemorrhagic versus non-hemorrhagic.

The SNR losses observed in dark-blood T2*-weighted CMR may be explained on the basis of the DIR preparation to attain the appearance of dark blood within the LV chamber. DIR preparation employs two adiabatic inversion pulses, which are applied at the R-wave of a heartbeat. Adiabatic RF pulses are longer than conventional RF pulses to ensure that the adiabatic condition (preservation of the direction of the effective magnetic field during a period of precession around effective field) is met. This can lead to significant loss of magnetization after inversion [23, 24], which is amplified when two adiabatic inversion pulses are used, as is the case with DIR preparations. This is consistent with our observations of lower SNR we observed with dark-blood T2*-weighted images compared to a bright-blood T2*-weighted images, where no DIR preparation is applied. This effect is more pronounced at 3 T compared to 1.5 T as the duration of the adiabatic inversion pulse is typically doubled at 3 T, which likely explains the greater SNR loss in dark-blood images at 3 T compared to 1.5 T.

Application of dark-blood prepared T2*-based CMR in the assessment of global iron overload in thalassemia has proven to be beneficial since it provided a means to improve the delineation of the boundary between the blood pool and the myocardium [25]. However, this does not appear to be the case in hemorrhagic MIs, where the iron comprising components of IMH are only found in focal MI regions within the myocardium. Notably, IMH which appears as a hypointense core in the myocardium emanates from the sub-endocardium, which can be incorrectly visualized as part of the blood pool appearing dark in images with the dark-blood preparation. Also, the appearance of dark blood pool within the LV chambers is premised on sufficient cardiac contraction to wash out the blood in the slice of interest, which then is replaced with blood that experienced the first (non-selective) inversion pulse in DIR preparation. In the setting of MI, infarcted walls have compromised contraction. This results in static or slow-moving blood at the infarct border to be only incompletely washed out with each heartbeat, thus giving the impression of a thicker wall. One important consequence of this is that it limits accurate border delineation between the myocardium and blood pool. The appearance of slow-moving blood at the MI zone to mimic tissue has been previously reported in cases where DIR pulses are used for T2-based acquisition in the heart and large blood vessels [26, 27]. These observations likely explain the weaker inter-observer reliability in dark-blood prepared T2*-weighted CMR compared to bright-blood T2*-weighted CMR.

One of the key findings from this study is that the size of IMH is significantly reduced in the dark-blood T2*-weighted images compared to bright-blood T2*-weighted images. The reduction in IMH size likely stems from reduced SNR and CNR due to DIR preparation. The loss of contrast between IMH and remote myocardium results in the underestimation of IMH Extent when quantification of hemorrhage is performed using the validated mean-2SD approach, since, by definition, this approach is sensitive to increased SD. Our observation here highlights an important limitation of dark-blood T2*-weighted CMR; that is, the reduction in IMH size implies that dark-blood T2*-weighted CMR can increase the false negatives of IMH, which is consistent with the observed significant reduction in sensitivity, accuracy and AUC compared to bright-blood T2*-weighted images. Another practical consequence of using dark-blood T2*-weighted approach is that the associated increase in false negatives with the approach would necessitate larger sample size for investigations aiming to modulate iron within MI in the pre-clinical and clinical settings.

Even though, the disparity between the findings in dark-blood versus bright-blood T2*-based CMR are smaller at 1.5 T, the negative impact of dark-blood technique on IMH detection is not negligible. One possibility to compensate for the reduction in SNR is to impose a significantly longer recovery time between DIR and readout. However, this has the added disadvantage of missing the blood-nulling point, ultimately compromising the value of dark-blood T2*-based approach. A second option is to reduce the adiabatic pulse duration, but this is likely to alleviate this problem at the cost of higher specific absorption rates, instead of solving it. Thus, more innovative approaches, for example employing modified adiabatic pulses, may be needed to improve the performance of dark-blood T2*-based CMR in the setting of non-invasive IMH detection. Until then, our findings support the notion that dark-blood and bright-blood images do not provide equivalent information with respect to IMH with the dark-blood T2*-based CMR carrying the risk of under diagnosing IMH. Hence, we recommend that among the T2* variants currently available, bright-blood T2*-based CMR should be the method of choice for identifying hemorrhagic MIs.

### Study limitations

Our study was limited to a small number of patients and animals as it was designed to evaluate the merits of dark-blood T2*-based approach against the conventional bright-blood T2*-based imaging in both clinical and preclinical settings [28]. Next, the findings from this study are only limited to the differences between bright- and dark-blood prepared T2*-based CMR. As such, our findings here do not reflect the existing limitations, particularly off-resonance issues compromising image quality in cardiac T2* imaging, that are also common to both bright- and dark-blood T2*-based imaging. Although the off-resonance issue may be mitigated by further innovations in shimming or imaging processing, such approaches are not yet available. Thus, we acquired both dark- and bright-blood T2* images with the state-of-the art shimming approaches currently available. To minimize the influence of these artifacts in this study, we (a) used only moderate TEs (~ 14 ms at 1.5 T and ~ 12 ms at 3 T) to balance image contrast against large signal voids; (b) studied both patients (24 of the 29) and animals (all) primarily with LAD infarctions to mitigate against prominent off-resonances artifacts at inferior and inferolateral walls [29]; and (c) excluded the inferior and inferolateral segments in image analysis when off-resonance artifacts were present. These efforts allowed us to selectively study the effects of dark-blood preparation while minimizing any confounding effects from off-resonance artifacts.

## Conclusions

While IMH can be visible on dark-blood T2*-weighted CMR, the overall conspicuity of IMH is significantly reduced compared to that observed in bright-blood T2*-weighted images, across infarct age in clinical and preclinical settings at 1.5 and 3 T. Hence, dark-blood T2*-weighted CMR should be used with the understanding that it carries the potential to misclassify hemorrhagic MIs as non-hemorrhagic MIs.

## Data Availability

Not applicable.

## Abbreviations

AHA: American Heart Association
CMR: Cardiovascular magnetic resonance
CNR: Contrast-to-noise ratio
COV: Coefficient of variations
DIR: Double inversion recovery
ECG: Electrocardiogram
IMH: Intramyocardial hemorrhage
LAD: Left anterior descending coronary artery
LCX: Left cirumflex coronary artery
LGE: Late gadolinium enhancement
LV: Left ventricle/left ventricular
MI: Myocardial infarction
MVO: Microvascular obstruction
PCI: Percutaneous coronary intervention
PSIR: Phase sensitive inversion recovery
RCA: Right coronary artery
ROI: Region-of-interest
SD: Standard deviation
SI: Signal intensity
SNR: Signal-to-noise ratio
STEMI: ST elevation myocardial infarction

## References

[1] Kloner RA (1993). Does reperfusion injury exist in humans?. J Am Coll Cardiol.

[2] Fishbein MC, Y-Rit J, Lando U, Kanmatsuse K, Mercier JC, Ganz W (1980). The relationship of vascular injury and myocardial hemorrhage to necrosis after reperfusion. Circulation.

[3] Olafsson B, Forman M, Puett D, Pou A, Cates C, Friesinger G, Virmani R (1987). Reduction of reperfusion injury in the canine preparation by intracoronary adenosine: importance of the endothelium and the no-reflow phenomenon. Circulation.

[4] Betgem RP, De Waard GA, Nijveldt R, Beek AM, Escaned J, Van Royen N (2015). Intramyocardial haemorrhage after acute myocardial infarction. Nat Rev Cardiol.

[5] Roberts CS, Schoen FJ, Kloner RA (1983). Effect of coronary reperfusion on myocardial hemorrhage and infarct healing. Am J Cardiol.

[6] Ganame J, Messalli G, Dymarkowski S, Rademakers FE, Desmet W, Van de Werf F, Bogaert J (2009). Impact of myocardial haemorrhage on left ventricular function and remodelling in patients with reperfused acute myocardial infarction. Eur Heart J.

[7] Kali A, Kumar A, Cokic I, Tang RL, Tsaftaris SA, Friedrich MG, Dharmakumar R (2013). Chronic manifestation of postreperfusion intramyocardial hemorrhage as regional iron deposition: a cardiovascular magnetic resonance study with ex vivo validation. Circ Cardiovasc Imaging.

[8] Eitel I, Kubusch K, Strohm O, Desch S, Mikami Y, de Waha S, Gutberlet M, Schuler G, Friedrich MG, Thiele H (2011). Prognostic value and determinants of a hypointense infarct core in T2-weighted cardiac magnetic resonance in acute reperfused ST-elevation-myocardial infarction. Circ Cardiovasc Imaging.

[9] Cokic I, Kali A, Yang HJ, Yee R, Tang R, Tighiouart M, Wang X, Jackman WS, Chugh SS, White JA, Dharmakumar R (2015). Iron-sensitive cardiac magnetic resonance imaging for prediction of ventricular arrhythmia risk in patients with chronic myocardial infarction: early evidence. Circ Cardiovasc Imaging.

[10] Mather AN, Fairbairn TA, Ball SG, Greenwood JP, Plein S (2011). Reperfusion haemorrhage as determined by cardiovascular MRI is a predictor of adverse left ventricular remodelling and markers of late arrhythmic risk. Heart.

[11] Behrouzi B, Weyers JJ, Qi X, Barry J, Rabadia V, Manca D, Connelly J, Spino M, Wood JC, Strauss BH (2020). Action of iron chelator on intramyocardial hemorrhage and cardiac remodeling following acute myocardial infarction. Basic Res Cardiol.

[12] Kali A, Tang RL, Kumar A, Min JK, Dharmakumar R (2013). Detection of acute reperfusion myocardial hemorrhage with cardiac MR imaging: T2 versus T2. Radiology.

[13] Kumar A, Green JD, Sykes JM, Ephrat P, Carson JJ, Mitchell AJ, Wisenberg G, Friedrich MG (2011). Detection and quantification of myocardial reperfusion hemorrhage using T2*-weighted CMR. JACC.

[14] Anderson L (2001). Cardiovascular T2-star (T2*) magnetic resonance for the early diagnosis of myocardial iron overload. Eur Heart J.

[15] He T, Gatehouse PD, Kirk P, Tanner MA, Smith GC, Keegan J, Mohiaddin RH, Pennell DJ, Firmin DN (2007). Black-blood T2* technique for myocardial iron measurement in thalassemia. J Magn Reson Imaging.

[16] Liguori C, Di Giampietro I, Pitocco F, De Vivo AE, Schena E, Mortato L, Pirro F, Cianciulli P, Zobel BB (2014). Dark blood versus bright blood T2* acquisition in cardiovascular magnetic resonance (CMR) for thalassaemia major (TM) patients: Evaluation of feasibility, reproducibility and image quality. Eur J Radiol.

[17] Messroghli DR, Moon JC, Ferreira VM, Grosse-Wortmann L, He T, Kellman P, Mascherbauer J, Nezafat R, Salerno M, Schelbert EB (2017). Clinical recommendations for cardiovascular magnetic resonance mapping of T1, T2, T2* and extracellular volume: a consensus statement by the Society for Cardiovascular Magnetic Resonance (SCMR) endorsed by the European Association for Cardiovascular Imaging (EACVI). J Cardiovasc Magn Reson.

[18] Kidambi A, Mather AN, Motwani M, Swoboda P, Uddin A, Greenwood JP, Plein S (2013). The effect of microvascular obstruction and intramyocardial hemorrhage on contractile recovery in reperfused myocardial infarction: insights from cardiovascular magnetic resonance. J Cardiovasc Magn Reson.

[19] O'Regan DP, Ahmed R, Karunanithy N, Neuwirth C, Tan Y, Durighel G, Hajnal JV, Nadra I, Corbett SJ, Cook SA (2009). Reperfusion hemorrhage following acute myocardial infarction: assessment with T2* mapping and effect on measuring the area at risk. Radiology.

[20] Bondarenko O, Beek AM, Hofman MB, Kühl HP, Twisk JW, Van Dockum WG, Visser CA, Van Rossum AC (2005). Standardizing the definition of hyperenhancement in the quantitative assessment of infarct size and myocardial viability using delayed contrast-enhanced CMR. J Cardiovasc Magn Reson.

[21] Kali A, Cokic I, Tang RL, Yang HJ, Sharif B, Marban E, Li D, Berman DS, Dharmakumar R (2014). Determination of location, size, and transmurality of chronic myocardial infarction without exogenous contrast media by using cardiac magnetic resonance imaging at 3 T. Circ Cardiovasc Imaging.

[22] O'Regan DP, Ariff B, Neuwirth C, Tan Y, Durighel G, Cook SA (2010). Assessment of severe reperfusion injury with T2* cardiac MRI in patients with acute myocardial infarction. Heart.

[23] Norris DG, Lüdemann H, Leibfritz D (1991). An analysis of the effects of short T2 values on the hyperbolic-secant pulse. J Magn Reson.

[24] Sorce DJ, Michaeli S, Garwood M (2007). Relaxation during adiabatic radiofrequency pulses. Curr Anal Chem.

[25] Smith GC, Carpenter JP, He T, Alam MH, Firmin DN, Pennell DJ (2011). Value of black blood T2* cardiovascular magnetic resonance. J Cardiovasc Magn Reson.

[26] Payne AR, Casey M, McClure J, McGeoch R, Murphy A, Woodward R, Saul A, Bi X, Zuehlsdorff S, Oldroyd KG, Tzemos N, Berry C (2011). Bright-blood T2-weighted MRI has higher diagnostic accuracy than dark-blood short tau inversion recovery MRI for detection of acute myocardial infarction and for assessment of the ischemic area at risk and myocardial salvage. Circ Cardiovasc Imaging.

[27] Simonetti OP, Finn JP, White RD, Laub G, Henry DA (1996). "Black blood" T2-weighted inversion-recovery MR imaging of the heart. Radiology.

[28] Triadyaksa P, Oudkerk M, Sijens PE (2020). Cardiac T2* mapping: Techniques and clinical applications. J Magn Reson Imaging.

[29] Positano V, Pepe A, Santarelli MF, Ramazzotti A, Meloni A, De Marchi D, Favilli B, Cracolici E, Midiri M, Spasiano A (2009). Multislice multiecho T2* cardiac magnetic resonance for the detection of heterogeneous myocardial iron distribution in thalassaemia patients. NMR Biomed.

